# Study on Moisture Phase Changes in Bread Baking Using a Coupling Model

**DOI:** 10.3390/foods14091649

**Published:** 2025-05-07

**Authors:** Luo Zhang, Wei Yang, Kai Xu, Linshuang Long, Hong Ye

**Affiliations:** Department of Thermal Science and Energy Engineering, University of Science and Technology of China, Hefei 230027, China; zhangluo@mail.ustc.edu.cn (L.Z.);

**Keywords:** bread baking, moisture phase change, heat and mass transfer, large deformation, coupled model

## Abstract

Moisture phase change (MPC), a key process in bread baking, significantly impacts heat and mass transfer, as confirmed by experiments. However, existing models poorly characterize this phenomenon, and its quantitative impact on baking needs systematic study. This research develops a coupled multiphase model for heat and mass transfer with large deformation, employing both equilibrium and nonequilibrium approaches to describe MPC in closed and open pores, respectively. Experimentally calibrated pore-opening functions and viscosity variations revealed that pore-opening primarily occurs at 71–81 °C, whereas dough solidification occurs at 50–110 °C. Model-based analysis indicates that in closed pores, evaporation–diffusion–condensation is the primary mode of moisture transport and heat transfer with contributing approximately 60% of the total effective thermal conductivity, and when pores open, water vapor evaporates or condenses on pore walls, forming an ‘evaporation front’ and ‘condensation front’. The content of liquid water increases at the ‘condensation front’ and decreases at the ‘evaporation front’. Bread deformation is predominantly governed by pressure differentials between closed pores and the ambient environment, with the partial pressure of water vapor emerging as the principal driver because its average content exceeds 70% within closed pores. These findings demonstrate that MPC governs heat and mass transfer and deformation during bread baking.

## 1. Introduction

Bread, one of the most important staple foods [1], undergoes complex physicochemical changes during thermal processing [2]. Bread has a porous structure composed of a matrix and pores [3]. The matrix contains both solid and liquid phases, while the pores are filled with gases, including water vapor and CO_2_ [4]. The solid phase is a network formed by starch and proteins, and the liquid water is embedded within this network through hydrogen bonds. Upon heating, heat is conducted inward, causing liquid water to diffuse and evaporate on the pore walls. As the temperature increases, dissolved CO_2_ escapes, expanding the pores and causing the bread to rise. When the pore walls rupture due to increasing gas pressure, the pores interconnect, facilitating gas flow driven by pressure differences. This reduces the gas pressure, while starch gelatinization, protein denaturation, and moisture loss gradually solidify the dough.

Bread has a porous structure, consisting of a matrix and pores [3]. The pores are filled with a gas phase that includes water vapor and CO_2_, whereas the matrix consists of both liquid and solid phases [4]. The solid phase is a network structure formed by starch and proteins [5,6], and the liquid phase water is embedded within this network through hydrogen bonds in the form of water molecules [7]. When the dough surface is heated, heat is conducted inward [8]. Liquid water diffuses in the matrix due to concentration gradients and evaporates on the pore walls [9]. As the temperature increases, dissolved CO_2_ escapes from the liquid phase [10]. This results in rapid pore expansion due to the increased amount of gas, as well as the rising temperature, causing the bread to expand [11]. When the closed-pore walls can no longer withstand the rapidly increasing internal gas pressure, they rupture [4], allowing the previously independent pores to interconnect and facilitating gas phase flow driven by pressure differences [12]. Upon pore opening, the gas pressure decreases, whereas starch gelatinization, protein denaturation, and moisture reduction contribute to the gradual solidification of the dough [13]. During high-temperature baking, Maillard reactions and caramelization occur on the outer surface of bread [14], imparting its characteristic brown color and flavor [15].

Building an accurate mathematical model to characterize the bread baking process is vital for enhancing baking techniques, and this area has been extensively researched. Zanoni [8] and Purlis [16] modeled heat and mass transfer within bread via effective thermal conductivity and effective diffusion coefficients, respectively, with the assumption that moisture evaporation occurs solely at the outer surface. These models can be used preliminarily to predict the temperature and mass changes during bread baking. However, these models cannot be used to reveal the internal physical transformations of bread and, thus, cannot be used for further analysis of the baking process. Vries [17] measured the internal temperature changes in porous and nonporous doughs and reported that porous dough heated more rapidly because the ‘evaporation–condensation’ process occurred within the pore structure. Wagner [18] experimentally reported an increase in water content at the crumb during bread baking, further confirming the occurrence of moisture phase changes within the bread. Their experiments confirmed moisture phase changes in bread and their significant impact on heat and mass transfer. Zhang [12] developed coupled heat and mass transfer equations for bread baking, modeling dough as a saturated porous medium. The equations account for liquid and gas phases, as well as bread deformation due to gravity and gas pressure. However, the gas phase pressure was determined empirically based on temperature and water content, omitting detailed physical processes and the effects of moisture phase change. Nicolas [19] developed a multiphase model to simulate heat and mass transfer, as well as deformation during bread baking, which successfully captured critical physical processes including pore formation and solidification. The rate of phase change formulation in the model, derived from mass conservation of liquid water, accounts for moisture loss through evaporation but neglects the internal evaporation–condensation dynamics that govern water redistribution within the bread matrix. Moreover, the gas pressure, a key driver of deformation, which is calculated by mass conservation of carbon dioxide, does not account for the effect of water vapor. Thus, the potential impact of water vapor on dough expansion and structural development during baking is ignored. This phenomenon means that the coupled mechanisms of heat and mass transfer and deformation within bread are not fully understood. This model accurately predicted water evaporation, pore formation, and dough solidification, enhancing the accuracy of temperature, moisture content, and shape change predictions. Lucas [20] proposed that, in addition to diffusion, water transport in bread occurs via an evaporation–condensation–diffusion mechanism within closed pores. He derived the transport flux based on mass conservation and revised the heat and mass transfer equations for the closed-pore region. However, the authors proposed that gas transport adheres to Darcy’s law in open pores, focusing solely on pressure-driven flow while neglecting the concurrent evaporation–condensation phase changes in water vapor. In recent years, research on the bread baking process has focused on energy savings, considering aspects such as heating methods [21] and oven structure design [22], health [23], and optimizing baking processes such as baking temperature [2], fermentation time [3], quality evaluation methods [24], and material formulation [25]. However, bread baking models undeniably remain the cornerstone of these studies.

In summary, although experiments have confirmed the significant impact of moisture phase changes on heat and mass transfer, existing models have not systematically analyzed this process. Due to the incomplete understanding of the role of moisture phase changes in baking in previous studies, the coupling relationships among heat transfer, mass transfer, and deformation have not yet been fully established. In this work, a coupled model integrating heat transfer, mass transfer, and deformation was developed to analyze moisture phase change and transport processes during bread baking. The model distinguishes between open and closed pore regions, employing equilibrium and nonequilibrium methods to describe moisture phase change mechanisms in these regions, respectively. The key parameters during bread baking were determined through experimental measurements, and the effects of moisture phase changes on heat transfer, mass transfer, and deformation were systematically analyzed via the model.

## 2. Experiment

### 2.1. Sample Preparation

Dough is made predominantly of wheat flour, water, and yeast. The W/F ratio is conventionally maintained between 0.5 and 0.6 [10,19,26], with a median value of 0.55 employed in this study. Following the yeast manufacturer’s guidelines, 1 g of yeast was added per 100 g of flour. The ingredients are mixed in the specified proportions via a dough mixer. After 10 min of mixing, the dough was subjected to 35 °C isothermal fermentation for 60 min. It is then remixed for an additional 10 min to release the gases generated during fermentation, followed by a final 10 min fermentation period to yield dough suitable for bread baking.

### 2.2. Experimental Protocols and Measurement Methods

**Figure 1:**
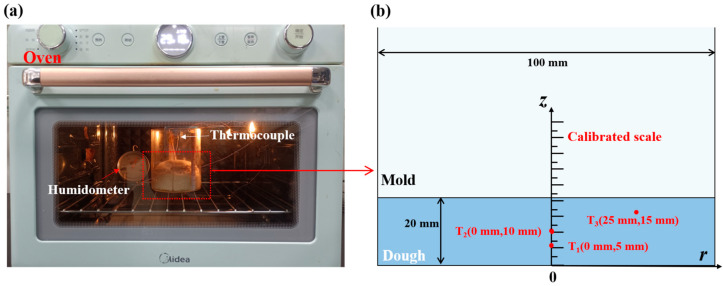
The experiment involves baking bread. (**a**) Experimental setup for bread baking. (**b**) Locations of the bread temperature measurement points and scale for bread height measurement.

An experimental setup was established to monitor the temperature, moisture content of the drying base, and height variations throughout the bread baking process, as depicted in Figure 1. To increase the visibility of the height changes in the bread, a transparent, heat-resistant borosilicate glass mold, as illustrated in Figure 1a, characterized by an inner diameter of 100 mm and a height of 100 mm, was employed in the study. The dough, initially measuring approximately 20 mm in height, was positioned within the mold. The mold was subsequently introduced into a preheated domestic oven set at 180 °C, with the baking duration fixed at 30 min. In Figure 1b, three internal temperature measurement points within the dough were designated T_1_ (0 mm, 5 mm), T_2_ (0 mm, 10 mm), and T_3_ (25 mm, 15 mm). These points were equipped with 0.2 mm diameter T-type thermocouples to track the internal temperature. The high flexibility of the thermocouples enabled them to conform to the expansion of the dough. Supplementary temperature sensors were deployed to gauge the mold wall, oven wall, and ambient air temperatures. A camera was synchronized to capture the bread’s expansion at 120 s intervals, documenting height changes at the peak of the bread’s top surface and the side edges of the bread’s top surface, as shown in Figure 1b. The experiment was replicated under consistent initial and boundary conditions to ensure repeatability, with data averaged across five trials. To evaluate the water content variations during baking, the dough samples were baked for 10, 20, or 30 min in separate trials, and mass measurements were taken after baking. The mass reduction was indicative of water loss, facilitating the calculation of the water content of the dough at different stages. To minimize measurement interference, temperature, height, and water content assessments were conducted in distinct experimental runs.

To ascertain the initial state of the dough, the initial moisture content was quantified via the direct drying method [27], a primary method known for its high detection accuracy, albeit being destructive and time-consuming. Additionally, the initial density was ascertained via the drainage method. The moisture state alterations and starch gelatinization are pivotal characteristics throughout the bread baking process. A differential scanning calorimeter (DSC, model 204 F1 Netzsch, Free State of Bavaria, Germany) was utilized to assess the moisture state and starch gelatinization of the dough within a temperature gradient of −40 °C to 100 °C, employing a heating rate of 10 °C/min [28], which is a standard procedure for such analyses [29].

## 3. Model

**Figure 2:**
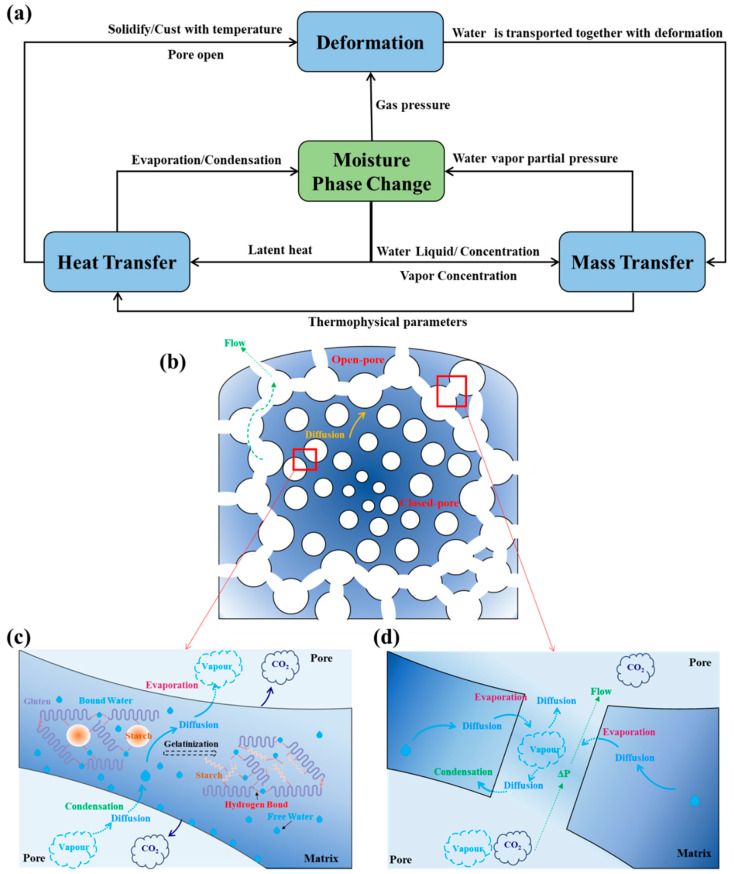
Schematic representation of the physical processes involved in bread baking. (**a**) Role of MPC in coupled mechanisms, (**b**) distribution of open-pore and closed-pore regions, (**c**) mass transfer process in closed-pore regions and the gelatinization of starch, and (**d**) mass transfer process in open-pore regions.

The dough is composed of three distinct phases, namely, solid, liquid, and gas, and each phase is treated as a continuous medium. Figure 2 illustrates the physical processes involved in the bread baking process.

In this physical model, the moisture phase change (MPC) is pivotal in linking heat transfer, mass transfer, and deformation processes, as depicted in Figure 2a. Heat transfer triggers phase transitions of internal moisture, thereby altering the moisture concentration and influencing mass transfer. The mass transfer of moisture not only modifies thermal properties such as thermal conductivity and specific heat, directly impacting heat transfer, but it also affects the extent of MPC, indirectly influencing heat transfer through latent heat changes. The MPC also alters the internal gas pressure, significantly affecting the deformation of the bread. Reciprocally, changes in the deformation process of bread impact the MPC. During baking, the pores of bread shift from a closed state to an open state, leading to substantial alterations in moisture transport. Initially, the interior of the bread is compartmentalized, as illustrated in Figure 2b, with water transport occurring via an evaporation–condensation–diffusion (ECD) mechanism. Specifically, liquid water in the matrix evaporates on pore walls to form water vapor, which fills the pores due to concentration gradients in closed pores. When water vapor encounters cooler walls, it condenses into liquid water and diffuses through the matrix, driven by concentration differences, as illustrated in Figure 2c [20]. Once the pores interconnect after opening the pores [4], water vapor, which is concentrated in areas with relatively high evaporation rates, is transported to areas of relatively low concentration under the influence of concentration gradients. The internal pressure of the bread exceeds the ambient pressure, prompting the release of water vapor and CO_2_ into the environment upon hole opening, as shown in Figure 2d. Water vapor also condenses upon encountering cooler walls during its transit through the pores.

### 3.1. Governing Equations

The distinct mass transfer mechanisms across the solid, liquid, and gas phases necessitate separate mass conservation equations for each phase. For the solid phase, the equation can be expressed as follows [30]

(1) $$\frac{\partial {\bar{\rho }}_{s}}{\partial t}+\nabla \cdot \left({\bar{\rho }}_{s}v\right)=0$$

where ${\bar{\rho }}_{s}$ denotes the apparent density of the solid phase, reflecting the mass of the solid components within a unit volume of bread. The second term on the left-hand side of the equation signifies the mass transport attributed to bread deformation, with **v** representing the velocity of this deformation.

The conservation equation for the water mass in the liquid phase is given by

(2) $$\frac{\partial {\bar{\rho }}_{l}}{\partial t}+\nabla \cdot \left({\bar{\rho }}_{l}v\right)+\nabla \cdot j_{l}={\dot{m}}_{l}$$

where ${\bar{\rho }}_{l}$ is the apparent density of the liquid phase, indicating the mass of the liquid phase per unit volume of bread. The second term on the left side of the equation signifies the mass transport due to liquid phase water, encompassing both deformation and the total diffusion flux. The total diffusion flux accounts for mass changes due to Fickian diffusion and the ECD mechanism, as delineated in

(3) $$j_{l}=j_{l,\mathrm{diff}}+j_{l,\mathrm{ECD}}$$

The liquid phase diffusion flux, $j_{l,\mathrm{diff}}$, is detailed in [31]

(4) $$j_{l,\mathrm{diff}}=-{\bar{\rho }}_{s}D_{l}\nabla W$$

where *W*, the water content (on a dry weight basis), is defined as the ratio between the mass of liquid water and the dry mass and where $D_{l}$ is the liquid water diffusion coefficient. In closed pores, water is transported via the ECD mechanism. As these pores are small and isolated, the water vapor inside quickly reaches saturation. Thus, evaporation here is considered balanced and instantaneous, following equilibrium equations. Mass conservation dictates that the water mass transfer flux in the matrix, arising from phase transitions, equates to the water vapor diffusion flux within the pore [20]. Therefore, the flux of the ECD can be expressed as

(5) $$j_{l,\mathrm{ECD}}=-f_{v,g}\alpha D_{v}\nabla C_{v}$$

where $f_{v,g}$ represents the volume fraction of the gas phase. Liquid water is adsorbed within the matrix and is not present in the pores. Hence, the porosity of the bread can be equated to the gas phase volume fraction. *α* represents the pore opening function, which quantifies the degree of pore opening in the bread, with a value ranging from 0 to 1. $D_{v}$ is the gas diffusion coefficient, and $C_{v}$ represents the water vapor concentration. Owing to their small size and isolation of closed pores, water vapor is assumed to have reached equilibrium prior to pore opening. The concentration gradient of water vapor within the pores is negligible, the concentration is at saturation levels [32,33], and the concentration can be formulated as

(6) $$C_{v}=a_{w}C_{\mathrm{sat}}=a_{w}\frac{P_{\mathrm{sat}}}{RT}$$

where *R* is the ideal gas constant, $a_{w}$ is the water activity, reflecting the strength of water binding to other molecules in a moist material, and is typically expressed as the ratio of the partial pressure of water vapor in the material to that of pure water [34]. The water activity can be determined by [35]

(7) $$100W=A{\left(\frac{a_{w}}{1-a_{w}}\right)}^{B}$$

here, $A=15.64-0.1\left(T-273.15\right)$ and $B=0.38+1.69\times 10^{-3}\left(T-273.15\right)$ [35]. By substituting Equation (6) into Equation (7), we obtain

(8) $$j_{l,\mathrm{ECD}}=\frac{-f_{v,g}\alpha D_{v}a_{w}}{RT}\nabla P_{\mathrm{sat}}$$

In Equation (2), the mass source term of the phase change signifies the variation in the liquid water mass due to MPC. In open pores, which are interconnected and in contact with the external environment, the water vapor concentration near the pore wall deviates from saturation due to vapor transfer driven by pressure gradients, rendering the equilibrium model inapplicable. The MPC is modeled via a nonequilibrium phase change approach [33], and the mass source term of the phase change is formulated as [32]

(9) $${\dot{m}}_{l}=k_{\mathrm{MPC}}f_{v,g}\left(a_{w}C_{\mathrm{sat}}-C_{v}\right)M_{H_{2}O}$$

where *k*_MPC_ represents the MPC rate constant, defined as the inverse of the time, s^−1^, required for the system to achieve equilibrium mass transfer [36].

Notably, $j_{l,\mathrm{ECD}}$ quantifies the water flux resulting from ECD between distinct volume elements. In contrast, ${\dot{m}}_{l}$ denotes the mass source of water within a volume element. While both involve water phase transitions, they represent different processes.

The gas phase, which includes CO_2_ and water vapor, involves distinct formation mechanisms and must be considered separately. The mass conservation equation for water vapor is presented in

(10) $$\frac{\partial {\bar{\rho }}_{v}}{\partial t}+\nabla \cdot ({\bar{\rho }}_{v}v+j_{v})=-{\dot{m}}_{l}$$

where the mass flux of water vapor, $j_{v}$, is expressed as follows:

(11) $$j_{v}={\bar{\rho }}_{v}u-\alpha f_{v,g}D_{v}\nabla C_{v}$$

where the first term on the right-hand side represents the convective mass flux of water vapor, with **u** being the gas phase velocity. The second term signifies the diffusive mass flux of water vapor. Notably, in closed pores, moisture transfer occurs exclusively via the EDC mechanism, with no interpore water vapor mass transfer occurring. The mass conservation equation for CO_2_ is given by

(12) $$\frac{\partial {\bar{\rho }}_{{\mathrm{CO}}_{2}}}{\partial t}+\nabla \cdot ({\bar{\rho }}_{{\mathrm{CO}}_{2}}v+j_{{\mathrm{CO}}_{2}})={\dot{m}}_{{\mathrm{CO}}_{2}}$$

where the mass flux of CO_2_, $j_{{\mathrm{CO}}_{2}}$, is detailed in

(13) $$j_{{\mathrm{CO}}_{2}}={\bar{\rho }}_{{\mathrm{CO}}_{2}}u-f_{v,g}\alpha D_{{\mathrm{CO}}_{2}}\nabla C_{{\mathrm{CO}}_{2}}$$

The mass source term for CO_2_, ${\dot{m}}_{{\mathrm{CO}}_{2}}$, can be expressed as [12,35]:

(14) $${\dot{m}}_{{\mathrm{CO}}_{2}}={\bar{\rho }}_{s}k_{{\mathrm{CO}}_{2}}f_{v,g}$$

where $k_{{\mathrm{CO}}_{2}}$ denotes the CO_2_ generation per kilogram of dry mass.

The gas phase flow is governed by [37]

(15) $$\rho_{g}\frac{\partial u}{\partial t}+f_{v,g}\rho_{g}\left(u\cdot \nabla u\right)=-\frac{\nabla P_{g}}{f_{v,g}}+\mu_{g}\nabla^{2}u-\frac{\mu_{g}}{\kappa }u$$

where the left-hand side’s first term denotes the inertial term, reflecting the rate of momentum change over time in the gas phase. The second term signifies the momentum associated with gas phase flow. On the right-hand side, the first term represents the momentum induced by pressure gradients, the second term accounts for momentum resulting from internal shear stress from gas viscosity, and the third term is the resistance term, indicating the drag force on the gas phase as it flows through pores. The gas phase density, $\rho_{g}$ (mass of gas per unit volume), is detailed in [19].

(16) $$\rho_{g}=\frac{P_{g}M_{g}}{RT}=\frac{\left(P_{v}+P_{{\mathrm{CO}}_{2}}\right)M_{g}}{RT}=\left(\frac{\rho_{v}}{M_{v}}+\frac{\rho_{{\mathrm{CO}}_{2}}}{M_{{\mathrm{CO}}_{2}}}\right)M_{g}$$

Bread deformation is typically modeled using solid mechanics [12,19], which requires mechanical parameters such as the elasticity modulus, relaxation time, and Poisson’s ratio. These complex parameters limit the practical application of the model. In contrast, fluid flow can describe material deformation using just equivalent viscosity as a mechanical property and has been successfully applied by Yang [30] and Lucas [38]. Initially, the dough behaves as a highly viscous fluid [20]. With decreasing moisture content and starch gelatinization and protein denaturation, the dough transitions to a solid state, characterized by increased resistance to deformation. The degree of viscosity reflects the ease of dough deformation. The momentum equation for the dough is given by

(17) $$\rho_{\mathrm{eff}}\left(\frac{\partial v}{\partial t}+v\cdot \nabla v\right)=-\nabla p+\nabla \cdot \tau +\rho_{\mathrm{eff}}g+F$$

where the left-hand side’s first term is the inertial term, with $\rho_{\mathrm{eff}}$ representing the dough’s equivalent density. The second term represents the momentum-associated dough deformation and flow. On the right-hand side, the first term denotes the pressure gradient-induced momentum, with *p* representing the effective pressure for dough flow. The second term represents the viscous momentum, where the Newtonian fluid’s viscous stress tensor, **τ**, is linearly related to the strain rate tensor, following the constitutive equations for Newtonian fluids [38]

(18) $$\tau =\mu_{\mathrm{eff}}\left(\nabla v+{\left(\nabla v\right)}^{T}-\frac{2}{3}\nabla \cdot vI\right)$$

The contribution of gas pressure to deformation is considered in the form of volume forces as

(19) $$F=-\nabla P_{g}$$

Heat transfer involves various modes, including deformation, flow, conduction, and latent heat transfer during bread baking. Under the assumption of local thermal equilibrium within the dough, the energy conservation equation for the porous medium is formulated as

(20) $${\left(\rho c_{p}\right)}_{\mathrm{eff}}\frac{\partial T}{\partial t}+\nabla \cdot \left({\left(\rho c_{p}\right)}_{\mathrm{eff}}vT+\rho_{g}c_{p,g}uT\right)=\nabla \cdot \left(\lambda_{\mathrm{eff}}\nabla T\right)-{\dot{m}}_{l}L_{v}$$

This equation is applicable to both open and closed pores. Notably, the EDC mechanism in closed pores contributes to energy transport. The effective thermal conductivity associated with the EDC mechanism captures the impact of latent heat changes on total heat transport. Furthermore, the MPC model differs between open and closed pores, necessitating distinct considerations for the mass source term of the phase transition, as detailed in Section 3.3.

### 3.2. Boundary and Initial Conditions

The dough is positioned within a cylindrical mold, and the boundary conditions of the bottom and side are similar. To facilitate calculations, an axisymmetric two-dimensional model is utilized, with the axis of symmetry serving as the axisymmetric boundary. The boundaries of bread are classified into two categories: the bread/air interface and the bread/mold interface.

The boundary conditions for bread baking, which include heat transfer, mass transfer, and deformation, can be categorized into solid, liquid, and gas phases. At the bread/air interface, the solid phase exhibits a no-flux condition, precluding mass transfer, whereas the liquid phase undergoes mass transfer through natural convection when the surface pores are closed. Once the surface pores open, liquid water evaporates into water vapor on the wall of the pores, transferring mass to the environment. The boundary condition of mass transfer for liquid water is given by

(21) $$j_{l,b}=\left(1-\alpha \right)h_{m}M_{H_{2}O}\left(a_{w}C_{\mathrm{sat}}-C_{\mathrm{oven},v}\right)$$

where *h*_m_ represents the natural convection mass transfer coefficient, which can be derived analogously to the natural heat transfer coefficient [31]. $C_{\mathrm{oven},v}$ represents the water vapor concentration in the oven, which can be measured via a hygrometer. The boundary conditions for mass transfer of water vapor follow natural convection when surface pores open, as detailed in

(22) $$j_{v,b}=\alpha h_{m}M_{H_{2}O}\left(C_{v}-C_{\mathrm{oven},v}\right)$$

The mass transfer boundary for CO_2_ is specified in

(23) $$j_{{\mathrm{CO}}_{2},b}=\alpha h_{{\mathrm{CO}}_{2}}M_{{\mathrm{CO}}_{2}}\left(C_{{\mathrm{CO}}_{2}}-C_{{\mathrm{oven},\mathrm{CO}}_{2}}\right)$$

where $h_{{\mathrm{CO}}_{2}}$ represents the natural convection mass coefficient for CO_2_, $C_{{\mathrm{CO}}_{2}}$ represents the CO_2_ concentration on the boundary surface, and $C_{{\mathrm{oven},\mathrm{CO}}_{2}}$ represents the CO_2_ concentration in the oven air. Under standard conditions, the mass concentration of CO_2_ in the air is approximately 8.6 × 10^−8^ kg/m^3^, and this value is considered negligible. The heat transfer boundary at the bread/air interface is expressed as follows:

(24) $$q^{\prime \prime }=h_{c}\left(T_{\mathrm{air}}-T\right)+\varepsilon \left[\Phi -\sigma T^{4}\right]-j_{l,b}L_{v}$$

where $h_{c}$ is the natural convective heat transfer coefficient, which is derived from experimental correlations [31]. The temperature of the air (*T*_air_) was experimentally determined. ε represents the emissivity of the bread surface, and Φ denotes the effective radiation density of the heating tube and top wall of the oven, which is averaged from foil radiation heat flow sensor (DF133-TRS-20) measurements at multiple positions near the bread mold. σ is the Stefan–Boltzmann constant. The deformation of bread at this interface is unrestricted, which marks it as a free boundary with ambient pressure.

At the bread/mold interface, given the dough’s tight adhesion to the mold wall, no mass transfer is assumed to occur at the interface. The bottom and side surfaces of the dough, in contact with the mold, transfer heat through conduction, with a heat flux of

(25) $$q=h_{e}\left(T_{w}-T\right)$$

where *h*_e_ is the contact heat transfer coefficient, which is preliminarily assigned a value of 100 W/(m^2^·K) [10]. *T*_w_ represents the wall temperature of the mold. The walls of the mold are so smooth that the friction between the bread and the walls is negligible. A no-slip boundary exists between the bread and the mold bottom, while a slip boundary with zero normal velocity is assumed between the bread and the mold side walls.

The initial conditions for the dough included a temperature of 28 °C, a moisture content (on a dry weight basis) of 0.82, and a density of 1069 kg/m^3^.

### 3.3. Parameters

The input parameters, including the component density, thermal conductivity, and diffusion coefficient, are listed in Table 1. Furthermore, several key parameters, including the MPC rate constant (*k*_MPC_), opening function (*α*), viscosity function (*η*), permeability (*κ*), and effective thermal conductivity ($\lambda_{\mathrm{eff}}$), should be discussed in detail.

The MPC rate constant is the reciprocal of the time of phase transition, which is used to characterize the speed of the water phase transition. The larger the value is, the faster the phase transition occurs [32], and it is a key parameter for calculating the distributions of water vapor and liquid water. In the bread baking experiment, the temperature of the crumb stabilized at 100 °C, indicating that the heat lost to water evaporation and the heat gained through conduction and convection had reached equilibrium, which suggests a lower limit for the *k*_MPC_. When the value of *k*_MPC_ increases, the convergence of the numerical solution of the equation gradually becomes worse [36]. For large *k*_MPC_ values (e.g., *k*_MPC_ > 5 s^−1^), equilibrium mass transfer is reached, and phase change occurs instantaneously [36], indicating an upper bound for *k*_MPC_. Notably, the closed pores conform to equilibrium mass transfer, with the MPC rate constant in closed pores (*k*_MPC, closed_) specified as 10 s^−1^ [32]. The open pores are characterized by nonequilibrium mass transfer. Thus, the MPC rate constant in open pores (*k*_MPC,open_) has small values (e.g., *k*_MPC,open_ < 5 s^−1^). Therefore, different MPC rate constants can separately describe the equilibrium mass transfer in closed pores and the nonequilibrium mass transfer in open pores, as detailed in

(26) $$k_{\mathrm{MPC}}=\alpha k_{\mathrm{MPC},\mathrm{open}}+\left(1-\alpha \right)k_{\mathrm{MPC},\mathrm{closed}}$$

**Figure 3:**
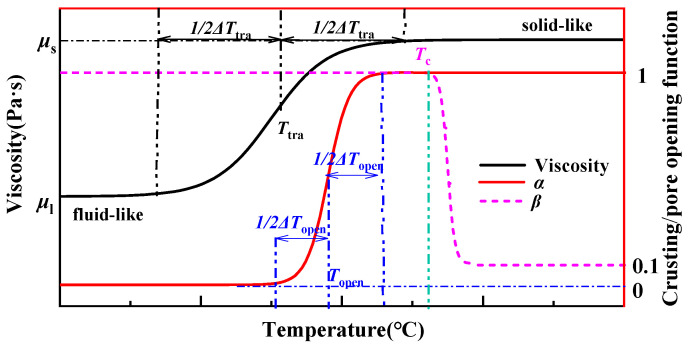
Pore opening function (*α*), crust function (*β*), and viscosity function (*μ*). *α* = 0 indicates closed pores, *α* = 1 indicates open pores, *β* = 1 represents crumbs, *β* = 0.1 represents crust, *μ*_l_ is the viscosity of fluid-like dough, and *μ*_s_ is the viscosity of solid-like dough.

Pore opening is a critical process during bread baking, as it alters the mass transfer modes of both the liquid and gas phases. Consequently, accurately describing the pore opening process is essential within the model. The mechanism of pore opening is highly complex [4], and the prevalent approach suggests that pores gradually open upon reaching a critical temperature threshold [19,35]. The opening function (*α*) is used to describe the opening process, as shown in Figure 3. The *α* is primarily dictated by the temperature of opening (*T*_open_) and the associated range of temperature (Δ*T*_open_). The value of *α* ranges from 0 to 1, with *α* = 0 for *T* < *T*_open_ − 1/2Δ*T*_open_, indicating closed pores, and *α* = 1 for *T* > *T*_open_ + 1/2Δ*T*_open_, indicating fully open pores. The values of *T*_open_ and Δ*T*_open_ are discussed in Section 4.1

In the closed-pore region, the gas phase remains stagnant, and it begins to flow following pore opening. Permeability is a crucial parameter in this flow process. During the baking process, the crust on the bread surface influences the state of gas phase flow. The permeability of the gas phase can be expressed as

(27) $$\kappa_{g}=\kappa_{0}\alpha \beta \phi^{1.34}$$

where ϕ denotes porosity, and since the liquid phase is bonded within the matrix, the porosity is equivalent to $f_{v,g}$. The crusting function (*β*) ranges from 1 to 0.1, reflecting that the permeability of gas in the crust is approximately an order of magnitude lower in the crumb after the bread has crusted [14]. Purlis [19] posited that the region with temperatures exceeding 100 °C delineates the bread crust; hence, *β* is illustrated in Figure 3.

The viscosity function (*μ*) characterizes the transition of dough from a fluid-like state to a solid-like state during the baking process. The viscosity of dough significantly increases due to starch gelatinization, protein denaturation, and moisture loss [20]. The sigmoid function captures the steep increase in dough viscosity. The *μ* is predominantly determined by the fluid-like viscosity (*μ*_l_) and solid-like viscosity (*μ*_s_), the transition temperature of the dough from fluid-like to solid-like (*T*_tra_), and the corresponding range of temperatures (Δ*T*_tra_), as shown in Figure 3. The viscosity of fluid-like dough is approximately 10^4^ Pa·s [20], and the viscosity of solid-like dough increases by two orders [10] of magnitude to approximately 4.5 × 10^6^ Pa·s [20]. Thus, the viscosity function requires the determination of *T*_tra_ and Δ*T*_tra_, as detailed in Section 4.1.

As a multicomponent material, the effective thermal conductivity of dough for heat transfer through conduction can be determined via the parallel model [32] as follows:

(28) $$\lambda_{\mathrm{eff},c}=f_{v,s}\lambda_{s}+f_{v,l}\lambda_{l}+f_{v,g}\lambda_{g}$$

Considering the mechanism of EDC within closed pores, the effective thermal conductivity of the EDC can be expressed as

(29) $$\lambda_{\mathrm{EDC}}\nabla T=-j_{l,\mathrm{EDC}}L_{v}=\frac{f_{v,g}D_{v}a_{w}L_{v}}{RT}\nabla P_{\mathrm{sat}}$$

(30) $$\lambda_{\mathrm{EDC}}=\frac{f_{v,g}D_{v}a_{w}L_{v}}{RT}\frac{dP_{\mathrm{sat}}}{dT}$$

Thus, the effective thermal conductivity of the dough during the baking process can be represented as

(31) $$\lambda_{\mathrm{eff}}=\lambda_{\mathrm{eff},c}+\left(1-\alpha \right)\lambda_{\mathrm{DEC}}f_{v,g}$$

### 3.4. Numerical Implementation

Using the finite element method, a 2D axisymmetric model was developed to simulate heat and mass transfer as well as deformation during the bread baking process. To ensure the accuracy and efficiency of the calculations, a sensitivity analysis was conducted on the quadrilateral mesh size. The maximum mesh sizes, number of elements, computation time of mesh (i) (where i = 1~4), and maximum deviations of the axis height and core temperature at steady state, and mass liquid water at 1800 s for mesh (i) compared with mesh (i-1) (where i = 2~4) are provided in Table 2. As the maximum mesh size decreases and the mesh count increases, mesh 4 has far more meshes than mesh 3 does, with the computation time being 4.4 times longer. The results from mesh 1 to mesh 3 show large deviations, but the results for mesh 3 and mesh 4 are quite close. Thus, mesh 3 was chosen to achieve the desired accuracy within the shortest computational time. Additionally, due to the significant temperature and concentration gradients at the boundaries [19], the boundary layer was refined to 0.1 mm. The transient fully coupled method was used to solve the system of equations, utilizing the direct solver PARDISO with the adaptive time stepping method and a relative tolerance of 0.1%. All the simulations were performed on a personal computer equipped with 16 GB of RAM and an Intel(R) Core (TM) i5-10400 CPU operating at 2.90 GHz. The simulation of the 1800 s baking process took approximately 1 h of computational time.

## 4. Results and Discussion

### 4.1. Determination of Key Parameters

*k*_MPC,open_, *T*_open_, Δ*T*_open_, *T*_tra_, and Δ*T*_tra_ are critical parameters in the bread baking model and significantly influence heat and mass transfer as well as the deformation process. These parameters, which are challenging to measure directly, are determined by fitting simulation results to experimental data concerning changes in temperature, mass, and height.

**Figure 4:**
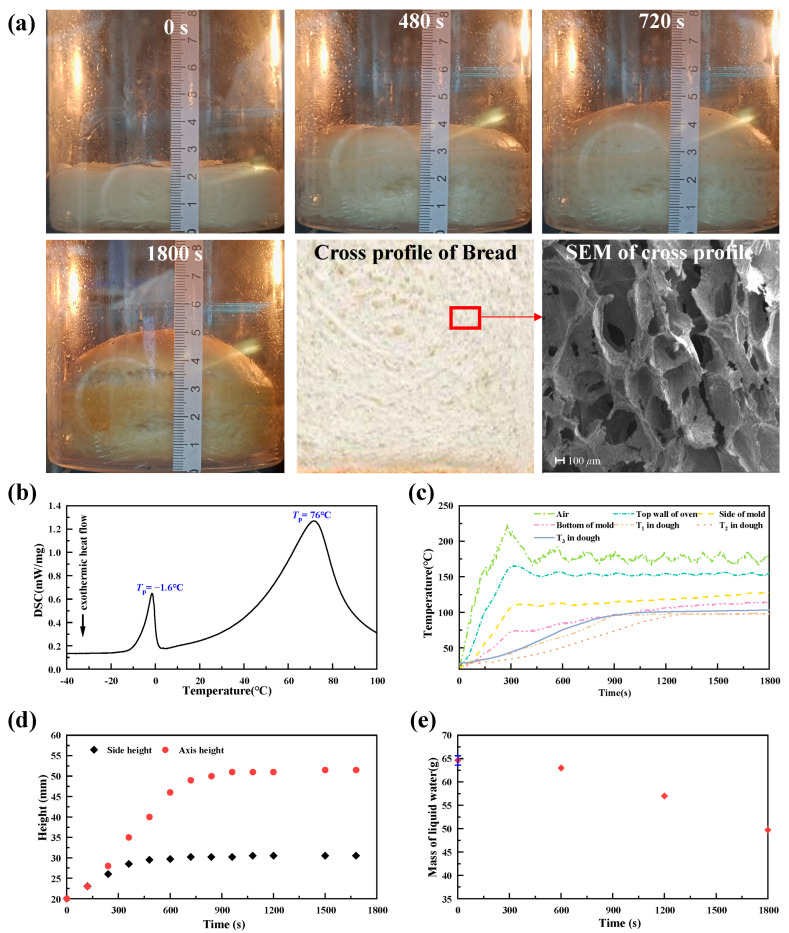
Bread baking experimental results. (**a**) Photographs of the bread at 0 s, 480 s, 720 s, and 1800 s, cross-sectional profile of the bread, and SEM results; (**b**) DSC curve of bread with respect to temperature; (**c**) temperature of the mold wall, ambient air, top wall of the oven, and T_1_/T_2_/T_3_ of the dough; (**d**) experimental results of the axis height and side height of the dough; (**e**) experimental results of the mass of liquid water.

Figure 4 presents the experimental results. Figure 4a shows the morphological changes in bread during baking. Initially, the center (axis) and edge (side) of the bread’s top surface rise synchronously. As baking progresses, the expansion at the side ceases gradually, whereas the axis height continues to rise until it stabilizes at a certain point. Ultimately, the color of the dough surface transitions to brown. Cross-sectional images and SEM analyses revealed the interconnected porous structure of the bread. Figure 4b shows the DSC curve of the dough, which presents two peaks between −40 °C and 100 °C. The first peak, at −1.6 °C, corresponds to the melting endotherm, indicating that the moisture in the dough is mainly bound water, with the major area of the peak below 0 °C [28]. The second peak, at 76.0 °C, is associated with starch gelatinization and water evaporation [29]. Figure 4c shows the temperatures of the mold wall, the oven’s air, the oven’s top wall, and the measuring points (T_1_, T_2_, T_3_) in the dough. Owing to intermittent oven heating, both the temperature of the oven’s air and the oven’s wall oscillate. The internal dough temperature incrementally reached a plateau at 100 °C. Figure 4d shows the height changes in the dough axis and side during baking. The side height stabilizes after approximately 450 s at approximately 30 mm, whereas the axis height stabilizes at approximately 900 s at approximately 51 mm. Figure 4e depicts the mass loss of liquid water, with an initial mass of 64.5 g and a loss of approximately 15.0 g by the end of the baking process.

Before parameter fitting, initial parameter estimations are essential. As analyzed in Section 3.3, the *k*_MPC,open_ is less than 5 s^−1^, with an initial assumption of 4 s^−1^. There is considerable variation in the reported *T*_open_ across different studies. Lucas et al. [32] reported *T*_open_ = 50 °C, with a Δ*T*_open_ of 10 °C, whereas Nicolas reported temperatures of 55 °C [19] and 65 °C [35]. Singh et al. [13] used a flooded parallel plate to monitor the rheological changes in the dough during baking. They observed an increase in the normal force exerted on the upper plate by the expanding dough, which was associated with dough expansion upon reaching 60 °C. There is a sudden drop in the normal force at 87 °C, suggesting the opening of the pores. Considering the possible correlation between pore opening and starch gelatinization, *T*_open_ was provisionally set to a gelatinization temperature of 76 °C in the DSC curve and Δ*T*_open_ = 10 °C [32]. *T*_tra_ was set as 65 °C in the literature [32], which is consistent with the findings of Singh et al. [13], who experimentally reported an increase in dough viscosity between 60 °C and 87 °C. Thus, *T*_tra_ can be preliminarily confirmed to be 70 °C, with Δ*T*_tra_ at 20 °C [32].

**Figure 5:**
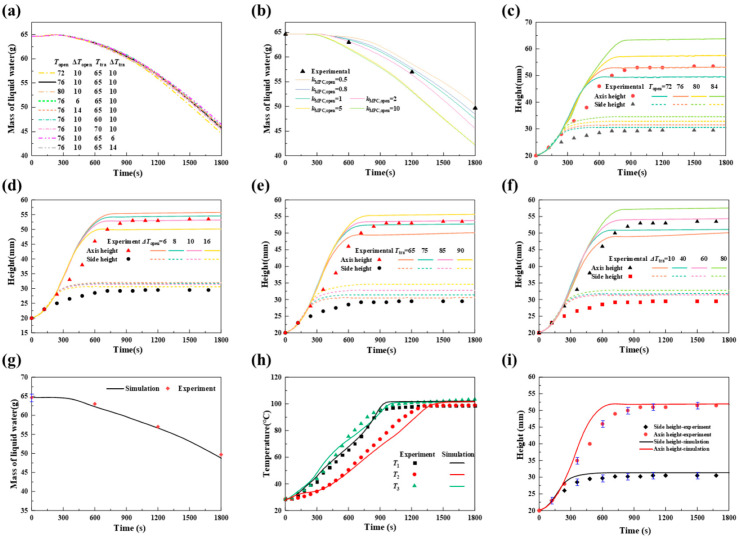
Parameter determination (**a**) Mass of liquid water changes with different combinations of *T*_open_, Δ*T*_open_, *T*_tra_, and Δ*T*_tra_; (**b**) mass of liquid water changes with different *k*_MPCvalues,opening_; (**c**) height of bread at varying *T*_open_; (**d**) height of bread at varying Δ*T*_open_; (**e**) height of bread at varying *T*_tra_; (**f**) height of bread at varying Δ*T*_tra_; and simulation results and experimental results of (**g**) mass of liquid water, (**h**) temperature, and (**i**) height with *k*_MPC,open_ = 0.8 s^−1^, *T*_open_ = 76 °C, Δ*T*_open_ = 10 °C, *T*_tra_ = 80 °C, and Δ*T*_tra_ = 60 °C.

The optimal parameter values were ascertained by comparing the numerical results with the mass of liquid water, height, and experimental temperature data. Figure 5 illustrates the results of the parameter determination. Preliminary precomputation revealed that the liquid water mass is insensitive to changes in *T*_open_, Δ*T*_open_, *T*_tra,_ and Δ*T*_tra_, as shown in Figure 5a. However, *k*_MPC,open_ significantly influences the liquid water mass. Therefore, the value of *k*_MPC,open_ can be determined by observing changes in the mass of liquid water in experiments. Figure 5b shows the changes in liquid water mass for various values of *k*_MPC,open_ (0.5 s^−1^, 0.8 s^−1^, 1 s^−1^, 2 s^−1^, 5 s^−1^, and 10 s^−1^). Observations indicate that as the value of *k*_MPC,open_ increases, the liquid water mass decreases more rapidly and significantly. This is due to the increased evaporation of liquid water into water vapor, which is then transported to the environment. Compared with the experimental measurements, the value of *k*_MPC,open_ is approximately 0.8 s^−1^. Additionally, when *k*_MPC,open_ exceeds 5 s^−1^ and continues to increase, the liquid water mass remains unchanged, which is consistent with findings in the literature [36].

Similarly, preliminary calculations have indicated that bread deformation is more sensitive to *T*_open_, Δ*T*_open_, *T*_tra_, and Δ*T*_tra_. Therefore, the changes in the axis and side heights were calculated with respect to these parameters to determine their values. Figure 5c–f display the axis and side heights of the bread at various values of *T*_open_, Δ*T*_open_, *T*_tra_, and Δ*T*_tra_. The larger *T*_open_ is, the greater the bread expansion. This is because a higher opening temperature results in a longer time for the closed pores, leading to higher internal pressure. Similarly, a higher Δ*T*_open_ results in earlier opening, leading to smaller bread expansion. Conversely, larger values of *T*_tra_ and Δ*T*_tra_ delay dough solidification, extending the deformation time and increasing bread expansion. Good agreement between the experimental and calculated values was observed when *T*_open_ = 76 °C, Δ*T*_open_ = 10 °C, *T*_tra_ = 80 °C, and Δ*T*_tra_ = 60 °C. The opening and transition temperatures align with findings in the literature [13].

The model is computed via these parameters, and the results are compared with experimental data for the mass of liquid water, temperatures of the measurement points, and heights of the bread. The comparison results are shown in Figure 5g–i. Figure 5g shows the change in mass of liquid water in the dough. The maximum deviation between the calculated and measured results of the liquid phase water mass change is 1.9%. Figure 5h compares the calculated and measured temperatures of T_1_, T_2_, and T_3_. On the basis of the experimental results, the process of temperature change can be divided into a warming stage and an equilibrium stage. T_1_ and T_3_, which are close to the boundary, have significantly higher heating rates than T_2_. T_1_ and T_3_ reach equilibrium at approximately 900 s, whereas T_2_ reaches equilibrium at approximately 1300 s. The equilibrium temperature is 100 °C, as this is when the evaporation rate is at a maximum, causing the water to evaporate and remove a large amount of heat. The core temperature of bread exceeds 93 °C at approximately 1020 s, indicating that the bread is fully baked [1]. The temperature of T_3_ continued to rise after 1600 s, which was likely due to the crust forming in this region. In the heating stage, T_3_ has a maximum deviation of 10.8%, whereas in the equilibrium stage, the deviations at each measurement point are less than 5%. Figure 5i shows the calculated and experimental results of the axis and side heights. During the initial 240 s, the dough expands uniformly, with the side height being approximately parallel to the axis height. The side height subsequently increases gradually and stabilizes at approximately 31 mm after approximately 600 s. The axis height continues to rise, reaching a maximum height of approximately 52 mm at approximately 600 s, followed by a decrease of approximately 0.2 mm. This decrease may be due to a minor collapse of the dough under gravity after full expansion. The deviation is significant during the rapid expansion stage, with the maximum deviation of the top center being approximately 16.5% and that of the edge height being approximately 5.4%.

The results demonstrate that the MPC rate constant is approximately 0.8 s^−1^ in the open pores. The pores of the dough gradually opened and interconnected between 71 °C and 81 °C. At approximately 50 °C, the viscosity of the dough increases, transitioning from a fluid-like state to a solid-like state. At approximately 110 °C, the dough solidifies completely because of water loss, starch dextrinization and protein coagulation. Based on these parameters, the model results for not only the weight loss of water and height change but also the measured temperature closely with the experimental data. This finding indicates that the model can describe heat transfer, mass transfer, and deformation during bread baking. With this model, we can analyze the physical changes within bread during baking in detail, particularly the effects of moisture phase changes on heat and mass transfer, as well as deformation.

### 4.2. Effect of the MPC on Heat Transfer

When the lowest temperature of the bread exceeds 93 °C, it is considered fully baked [1]. Based on the calculations, the bread reaches full maturity before 1200 s. Therefore, the analysis below focuses on the results calculated before 1200 s of baking.

**Figure 6:**
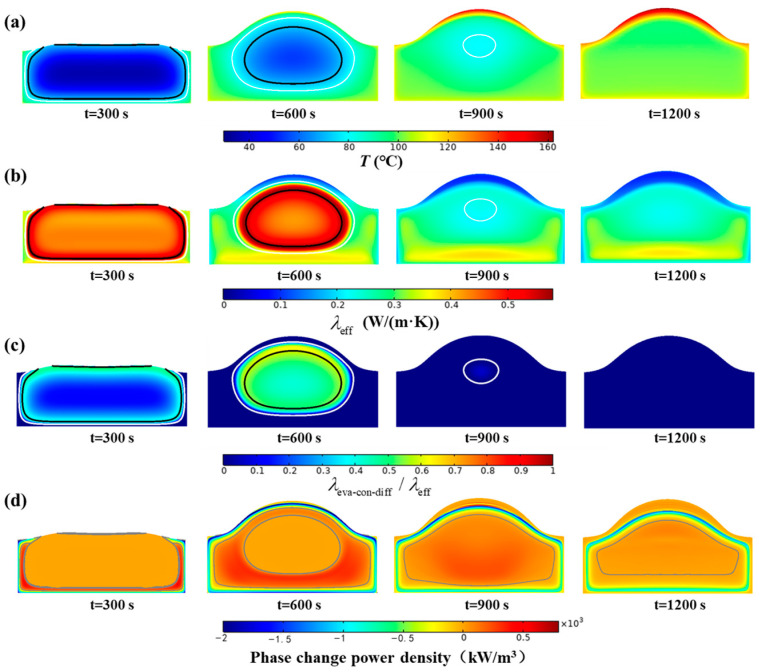
Effect of MPC on heat transfer: (**a**) temperature, (**b**) effective thermal conductivity, (**c**) ratio of EDC thermal conductivity to effective thermal conductivity, and (**d**) phase change power density. The solid black line and solid white line represent the contour lines of α = 0 and α = 1, respectively, and the solid gray line in (**d**) represents the contour lines where the phase change power density is zero.

Figure 6 illustrates the impact of the MPC on the heat transfer process. Figure 6a shows the temperature change in the bread at 300 s, 600 s, 900 s, and 1200 s, with the white and black lines representing the isotherms of *T* = 81 °C and *T* = 71 °C, respectively, indicating the critical points where the pores are fully open (*α* = 1) and fully closed (*α* = 0). A distinct temperature boundary is observable between the regions of closed pores and open pores, primarily due to their different heat transfer rates. Hence, Figure 6b shows the variations in the effective thermal conductivity of the bread over time. The effective thermal conductivity in the closed-pore region is significantly greater than that in the open-pore region, indicating that the EDC mechanism greatly enhances heat transport, which becomes ineffective once the pores open. The effective thermal conductivity decreases in the crust but is greater in the bottom region because of differences in moisture content. Figure 6c presents the ratio of the effective thermal conductivity due to the EDC mechanism to the total effective thermal conductivity to assess the influence of the EDC mechanism on heat transfer. As the temperature increases, the proportion of the effective thermal conductivity attributed to the EDC mechanism in the closed-pore region gradually increases and exceeds 60%. This indicates that the EDC mechanism becomes the primary mode of heat transfer in the closed-pore region. The MPC within the closed pores influences heat transfer through the EDC mechanism. In the open pores, water vapor condenses on the pore wall, which also impacts heat transport. Figure 6d shows the variations in the power density of the phase change during the bread heating process. The solid gray line is the contour line where the phase change power density is zero, which divides the entire area into evaporation and condensation regions. Positive values indicate heat gain, which corresponds to the moisture condensation process, whereas negative values indicate heat loss, which corresponds to the moisture evaporation process. An ‘evaporation front’ forms in bread with a high power density of evaporation and gradually moves inward. Additionally, there is a concentrated area of high condensation power density in the open-pore region, which also moves gradually inward, as the gray line shows, and can be termed the ‘condensation front’. The presence of the ‘condensation front’ accelerates heat transfer in the open pores, resulting in a rapid temperature increase. This also explains the distinct temperature boundary between the open pores and closed pores in Figure 6a. These two typical ‘fronts’ indicate that heat transfer caused by the MPC plays a dominant role in the overall heat transfer process.

### 4.3. Effect of the MPC on Mass Transfer

**Figure 7:**
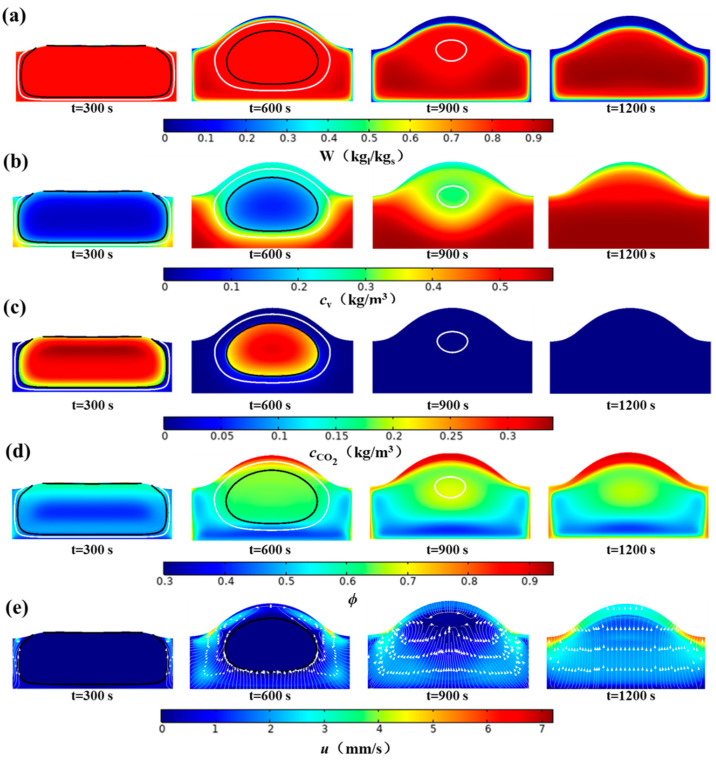
Effect of MPC on mass transfer (**a**) water content, (**b**) water vapor concentration, (**c**) CO_2_ concentration, (**d**) porosity, and (**e**) gas velocity distributions. (The solid black lines and solid white lines in (**a**–**d**) represent the contour lines of α = 0 and α = 1, respectively. The white arrows indicate the direction of gas flow).

Figure 7 illustrates the impact of MPC on the mass transfer process. Mass transfer primarily involves the transport of liquid and gas phases, with the MPC serving as the bridge between heat and mass transfer. Figure 7a shows that as baking progresses, the bread surface loses moisture due to evaporation, becoming dry and forming a crust. In the crumb, the open pores experience an increase in liquid water content due to the condensation of water vapor, even surpassing the initial concentration. Figure 7b indicates that in the closed pores, the water vapor concentration is saturated, whereas in the open pores, there is a gradient in the water vapor concentration that decreases gradually toward the environment. Figure 7c shows that CO_2_ is concentrated in the closed pores and is rapidly transported to the environment once the pores open, leaving no CO_2_ in the bread when the pores open fully. Figure 7d illustrates the change in the porosity of the bread. After solidification, the porosity in the closed-pore region gradually increases, and the bread core remains the most porous area, excluding the crust. The porosity of the crust slightly decreases during baking, and the bottom area of the bread has a lower porosity because of slight collapse under gravity after the pores fully open. Figure 7e shows the gas flow velocity within the bread, with white arrows indicating the direction of gas flow. In the open-pore region, the gas phase flows with increasing velocity as the area expands. Once the pores are fully open, the gas phase flows upward from the bottom to the environment.

**Figure 8:**
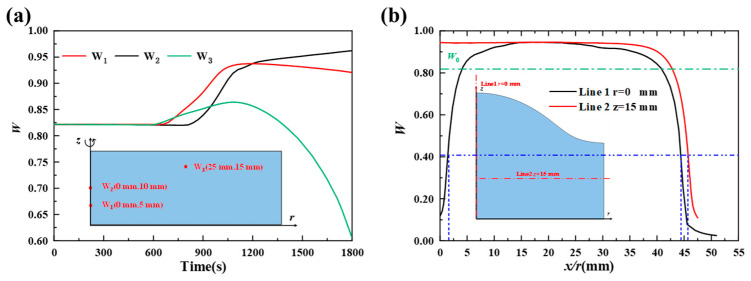
Spatial and temporal variations in water content at representative locations. (**a**) water content at W_1_/W_2_/W_3_ as a function of time, and (**b**) water content at lines 1/2.

Figure 8 denotes the spatial and temporal variations in the water content at representative locations. Figure 8a shows the variations in water content over time for points W_1_ (0 mm, 5 mm), W_2_ (0 mm, 10 mm), and W_3_ (25 mm, 15 mm). Initially, the water contents at these points remain relatively constant, indicating minimal moisture transport in the closed pores. After the pores open, water vapor condenses, causing a rapid increase in water content at all points. As the ‘evaporation front’ moves inward, water evaporates rapidly, and the water content decreases sharply until a crust forms. Figure 8b shows the dry basis water content distribution at x = 0 mm and z = 15 mm at 1200 s. The water content in the crumb can reach approximately 0.95, a 15.8% increase on a dry basis and 3.6% increase on a wet basis. Wagner et al. [18] experimentally measured the center moisture content (wet basis) of bread crumb during baking and reported a maximum increase of 1.3% (wet basis) between 4 and 7 min. This value is lower than those reported by Vries (8%) [17] and Thorvaldsson(18.7%) [41]. The discrepancy can be attributed to differences in baking time selected for observation and size of the dough. The driving force for water transfer toward the core is more intense and persistent for larger bread [18], resulting in a higher core moisture content. The crumb moisture varies with baking time, as shown in Figure 8a. This means that later observation times and larger dough sizes tend to result in greater moisture content increases in the crumb. In this study, the dough size used was smaller than that used in Wagner’s experiments, and the observation time was longer. Based on previous literature, a moisture content increase of 3.6% is entirely reasonable. The water content in the crust decreases rapidly. Apart from the crust, the water content distribution with the crumb is relatively uniform.

These phenomena indicate that MPC not only significantly influences the flow of gas and distribution of water vapor and CO_2_ but also affects the distribution of liquid water and porosity, which are key parameters related to the final quality of bread.

### 4.4. Effects of MPC on Deformation

**Figure 9:**
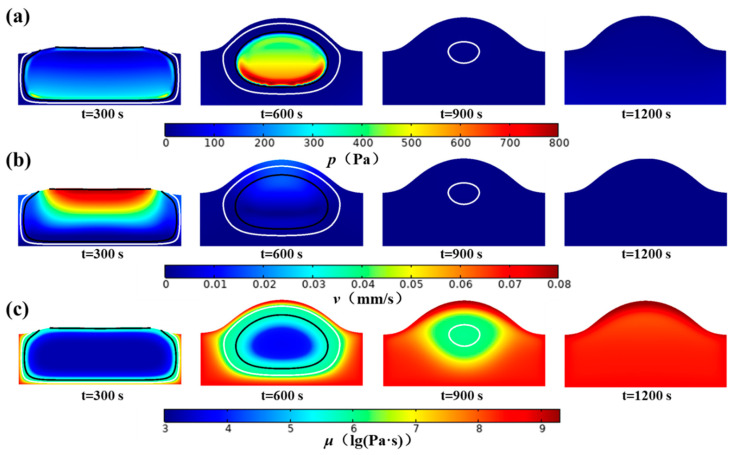
Effect of MPC on deformation (**a**) pressure, (**b**) deformation velocity, and (**c**) viscosity.

Figure 9 illustrates the impact of the MPC on deformation. The driving force behind bread deformation is the pressure difference between the gas in the bread and the ambient environment. Figure 9a shows the change in pressure difference between the air within the substrate and the ambient environment at different times. The pressure in the closed pores is the main driving force, and the pressure difference tends to 0 after the pore is opened. Figure 9b shows the deformation rate of bread under the combined influence of pressure difference and viscosity. The expansion rate is highest in the closed-pore region, and the bread ceases to expand after the pores are fully opened. Figure 9c shows the change in dough viscosity. As the temperature increases, the viscosity of bread increases, making deformation more difficult.

**Figure 10:**
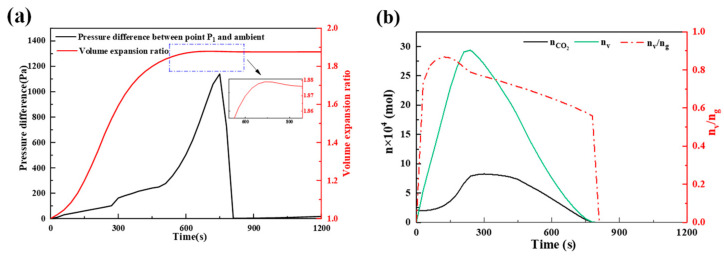
Volume expansion of bread. (**a**) bread volume expansion ratio and pressure difference between P_1_ and the ambient environment with time, and (**b**) the amount of water vapor and CO_2_ in the closed hole area and its ratio.

Figure 10 illustrates the changes in key parameters during dough expansion. Figure 10a shows the change in pressure difference between P_1_ (0 mm, 10 mm) and the environment, as well as the initial volume expansion ratio, compared with the volume of the bread. The maximum pressure of P_1_ can reach 1100 Pa, peaking at approximately 750 s before rapidly decreasing to ambient pressure due to pore opening. The bread’s final volume can expand to approximately 1.9 times its initial state. At approximately 750 s, the bread volume slightly decreases, which is attributed to gravitational collapse after the pores are fully opened. The results show the same trends and have similar parameter values to the experimental data from Nicolas [35]. Figure 10b shows the amount of water vapor and CO_2_ substances in the closed-pore region, as well as the proportion of water vapor in the gas phase. In the same environment, the ratio of these two substances can represent their contribution to the pressure difference. The proportion of water vapor increased rapidly, reaching 80% within 100 s, with an average of approximately 70% before opening. This indicates that water vapor is the primary gas phase component in the closed pores, meaning that its generation is the main factor driving bread expansion. This finding overturns the previous conclusion that water vapor contributes little to deformation [10,19].

## 5. Conclusions

A multiphase transport model was developed to elucidate the coupled heat and mass transfer and deformation mechanisms during bread baking. Pore development and viscosity changes were determined through experimental data fitting. The pore-opening process predominantly occurs within the temperature range of 71–81 °C, while dough solidifies from approximately 50 °C to 110 °C. In closed pores, the evaporation–diffusion–condensation (EDC) mechanism dominates heat transfer (contributing more than 60% of the effective conductivity) and water transport, with water vapor constituting 70% of the gas phase and driving expansion through its partial pressure. Following pore opening, vapor migrates via pressure–concentration gradients, accompanied by liquid water redistribution between evaporation and condensation fronts. These findings confirm that moisture phase change (MPC) is the central process governing thermal, hydraulic, and mechanical behaviors. The model is limited by its assumption of constant thermophysical properties. Future studies should investigate how temperature dependence, starch gelatinization, and protein denaturation influence these material characteristics.

## Figures and Tables

**Table 1:** Parameters used in the model.

Parameter	Value	Source	Parameter	Value	Source
$\rho_{\mathrm{eff},0}$	1069 kg/m^3^	Experiment	$c_{p,g}$	2030 J/kg	[39]
$\eta_{\mathrm{eff}}$	0.9	Experiment	$c_{p,l}$	4180 J/kg	[39]
Φ	1000 W/m^2^	Experiment	*T_c_*	100 °C	[9]
$T_{0}$	28 °C	Experiment	$\rho_{s}$	1500 kg/m^3^	[12,19]
$\rho_{l}$	1000 kg/m^3^	[12,19]	$\mu_{g}$	1.8 × 10^−5^ Pa·s	[31]
*E*_a_	138 kJ/mol	[40]	$\kappa_{0}$	8.77 × 10^−11^	[19]
*k*_g,0_	2.8 × 10^18^ s^−1^	[40]	*g*	9.8 m/s^2^	
$L_{v,\mathrm{ref}}$	2.454 × 10^6^ J/kg	[39]	*R*	8.314 J/(mol·K)	
*μ*_l_	10^4^ Pa·s	[20]	*μ*_s_	4.5 × 10^6^ Pa·s	[20]
*p*_sat_	$597\exp\left(\frac{T-273.15}{T-35.86}\right)$				[35]
$D_{l}$	$10^{-9}\varepsilon_{g}\exp\left(-10+10W\right)$				[35]
$D_{\mathrm{eff},g}$	$D_{g,i}{\varepsilon_{g}}^{4/3}$				[35]
*L*_v_	$L_{v,\mathrm{ref}}+\left(c_{p,g}-c_{p,l}\right)\left(T-T_{0}\right)$				[39]
$k_{{\mathrm{CO}}_{2}}$	$\begin{array}{c} 5\times 10^{-6}T-9.98\times 10^{-5}\;T\leq 40\;{}^{\circ}C\\ 10^{-4}\exp{\left(-\frac{T-40}{10}\right)}^{2}\;T>40\;{}^{\circ}C \end{array}$				[19]

**Table 2:** Mesh characteristics.

Mesh (i)	Maximum Mesh Size (mm)	Number	Time	Maximum Deviation
Mesh 1	1.25	1200	26 min	——
Mesh 2	0.75	1353	33 min	3.0%
Mesh 3	0.5	4738	47 min	4.1%
Mesh 4	0.3	11,122	207 min	0.3%

## Data Availability

The original contributions presented in this study are included in the article. Further inquiries can be directed to the corresponding authors.

## Nomenclature

D	water (liquid or vapor) or CO_2_ diffusion coefficient, m^2^ s^−1^
*W*	dry base liquid water content, kg kg^−1^
*M*	molar mass
*P*	pressure, Pa
*T*	temperature, K
*k*_MPC_	moisture phase change rate constant
*c*_p_	specific heat, J kg^−1^ K^−1^
*h*	mass transfer coefficient, m s^−1^
*h_c_*	convective heat transfer coefficient, W m^−2^ K^−1^
*L*_v_	latent heat, J kg^−1^
*C*	molar concentration of a species, mol m^−3^
**v**	velocity, m s^−1^
*R*	universal gas constant, J K^−1^ mol^−1^
**u**	velocity of gas flow
*g*	gravitational acceleration, m s^−2^
**F**	body force, N
**I**	unit tensor
*a_w_*	water activity
*f*_v_	volumetric fraction
***Greek symbols***	
*ρ*	density, kg m^−3^
$\bar{\rho }$	Phase apparent density, kg m^−3^
ϕ	porosity
*ε*	emissivity
*κ*	intrinsic/relative permeability, m^−2^
*σ*	stress
***τ***	strain rate tensor
*λ*	thermal conductivity, W m^−1^ K^−1^
*α*	pore opening function
*β*	crust function
*μ*	viscosity function
***Subscripts/Superscript***	
0	initial
l	liquid water
s	solid
g	gas
eff	effective
v	water vapor
sat	saturation
ref	reference
eva	evaporation
con	condensation
diff	diffusion
